# Crystallographic study of PET radio­tracers in clinical evaluation for early diagnosis of Alzheimers^1^

**DOI:** 10.1107/S1600536814021400

**Published:** 2014-10-04

**Authors:** Angela Altomare, Elena Capparelli, Antonio Carrieri, Nicola A. Colabufo, Anna Moliterni, Rosanna Rizzi, Dritan Siliqi

**Affiliations:** a Istituto di Cristallografia, Via G. Amendola, 122/o, 7016, Bari, Italy; b Dip. di Farmacia-Scienze del Farmaco, Universita’ degli studi di Bari, Via Orabona, 4, 70125, Bari, Italy; c Dip. di Farmacia-Scienze del Farmaco, Biofordrug, srl, Universita’ degli studi di Bari, Via Orabona, 4, 70125, Bari, Italy

**Keywords:** crystal structure, ligands, P-glycoprotein inhibitor, PET radiotracer, hydrogen bonds

## Abstract

The title compound, C_24_H_25_NO_3_·2CH_3_OH, which crystallized as a methanol disolvate, has applications as a PET radiotracer in the early diagnosis of Alzheimer’s disease. The dihedral angle between the biphenyl rings is 8.2 (2)° and the heterocyclic ring adopts a half-chair conformation with the N atom adopting a pyramidal geometry (bond-angle sum = 327.6°). The C atoms of both meth­oxy groups lie close to the plane of their attached ring [deviations = 0.107 (6) and 0.031 (6) Å]. In the crystal, the components are linked by O—H⋯O and O—H⋯N hydrogen bonds, generating [010] chains. C—H⋯O inter­actions are also observed.

## Related literature   

For pharmacological and biological studies of the title compound, see Colabufo *et al.* (2008 ▶, 2009 ▶).
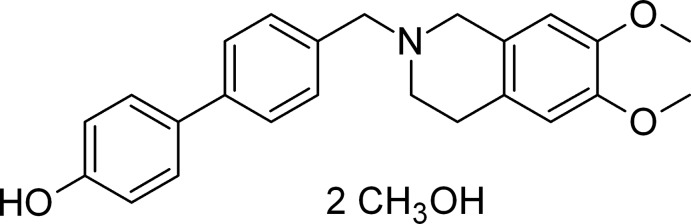


## Experimental   

### Crystal data  

C_24_H_25_NO_3_·2CH_4_O
*M* *_r_* = 439.53
Monoclinic, 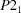

*a* = 8.894 (2) Å
*b* = 13.7187 (16) Å
*c* = 10.680 (2) Å
β = 111.575 (17)°
*V* = 1211.8 (4) Å^3^
*Z* = 2
Mo *K*α radiation
μ = 0.08 mm^−1^
*T* = 293 K
0.30 × 0.30 × 0.15 mm

### Data collection  

Bruker–Nonius KappaCCD diffractometer
Absorption correction: multi-scan (*SADABS*; Sheldrick, 2008*a* ▶) *T*_min_ = 0.921, *T*_max_ = 0.988
14813 measured reflections
5436 independent reflections
2610 reflections with *I* > 2σ(*I*)
*R*_int_ = 0.116

### Refinement  

*R*[*F* ^2^ > 2σ(*F* ^2^)] = 0.061
*wR*(*F* ^2^) = 0.115
*S* = 0.96
5436 reflections
305 parameters
1 restraint
H atoms treated by a mixture of independent and constrained refinement
Δρ_max_ = 0.17 e Å^−3^
Δρ_min_ = −0.15 e Å^−3^

### 

Data collection: *COLLECT* (Nonius, 2002 ▶); cell determination and refinement: *DIRAX* (Duisenberg,1992 ▶; Duisenberg *et al.*, 2000 ▶); data reduction: *EVAL* (Nonius, 2002 ▶; Duisenberg *et al.*, 2003 ▶); program(s) used to solve structure: *SIR2011* (Burla *et al.*, 2012 ▶); program(s) used to refine structure: *SHELXL2013* (Sheldrick, 2008*b* ▶); molecular graphics: *ORTEP-3 for Windows* (Farrugia, 2012 ▶) and *EXPO2013* (Altomare *et al.*, 2013 ▶); software used to prepare material for publication: *WinGX* (Farrugia, 2012 ▶) and *publCIF* (Westrip, 2010 ▶).

## Supplementary Material

Crystal structure: contains datablock(s) I, global. DOI: 10.1107/S1600536814021400/hb7266sup1.cif

Structure factors: contains datablock(s) I. DOI: 10.1107/S1600536814021400/hb7266Isup2.hkl

Supporting information file. DOI: 10.1107/S1600536814021400/hb7266Isup3.docx

**Fig. 1.**
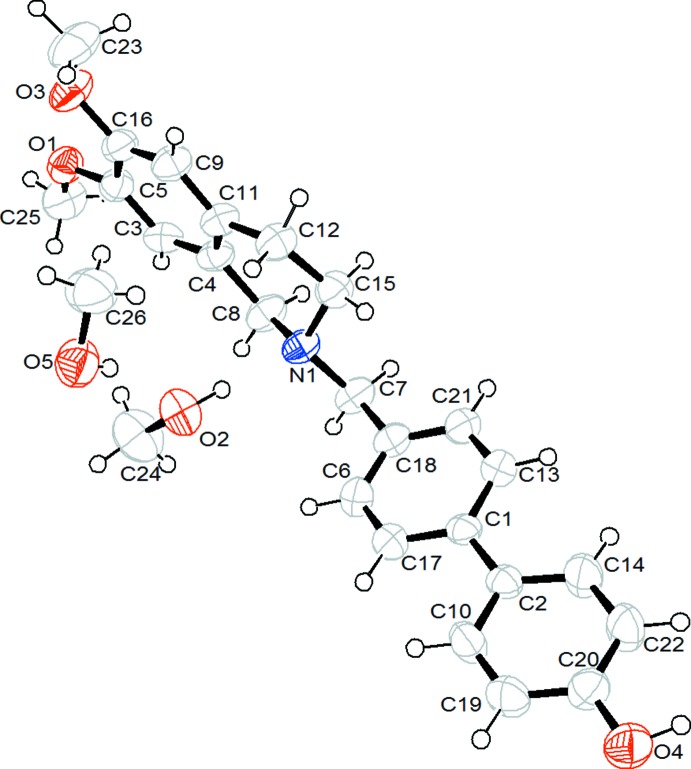
The molecular structure of the MC70 compound with displacement ellipsoids drawn at the 50% probability level.

. DOI: 10.1107/S1600536814021400/hb7266fig1.tif
The mol­ecular structure of the MC70 compound with displacement ellipsoids drawn at the 50% probability level.

**Fig. 2.**
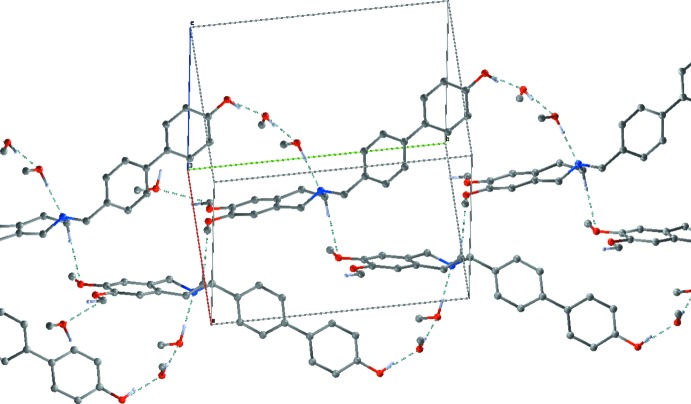
Crystal packing of the MC70 compound. The light blue dashed lines show the hydrogen bonds (see Table 1 for details).

. DOI: 10.1107/S1600536814021400/hb7266fig2.tif
Crystal packing of the MC70 compound. The light blue dashed lines show the hydrogen bonds (see Table 1 for details).

CCDC reference: 915609

Additional supporting information:  crystallographic information; 3D view; checkCIF report

## Figures and Tables

**Table 1:** Hydrogen-bond geometry (, )

*D*H*A*	*D*H	H*A*	*D* *A*	*D*H*A*
O2H2*O*N1	0.94(5)	1.87(5)	2.812(5)	178(4)
O4H4*O*O5^i^	0.95(6)	1.71(6)	2.636(6)	165(5)
O5H5*O*O2	0.71(8)	2.00(8)	2.684(6)	162(9)
C15H15*A*O1^ii^	0.97	2.50	3.445(6)	164
C23H23*A*O2^iii^	0.96	2.56	3.437(6)	152

Symmetry codes: (i) 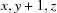; (ii) 
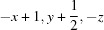
; (iii) 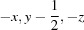.

## supplementary crystallographic information

### S1. Comment

The single-crystal X-ray structure solution of 4'-(6,7-dimethoxy-3,4-dihydro-1*H*-isoquinolin-2-yl-methyl)-biphenyl-4-ol (named MC70) radiotracer, previously pharmacologically characterized and biologically evaluated (compound 4e in Colabufo *et al.*, 2008, 2009), has been reported. At nanomolar concentrations MC70 is a potent inhibitor of P-glycoprotein (P-gp), a membrane protein playing a protective role of the central nervous system and whose numerical and functional alteration is responsible for the onset of the Alzheimer disease. The crystallographic characterization of MC70 represents the first necessary step for a further evaluation of its pharmacological properties and to obtain, f. e. through docking techniques and homology modelling, a tridimensional interpretation of the main molecular determinants responsible for most of MC70 features such as to be an inhibitor of the P-gp. The study of this behaviour will allow the design of new ligands, more effective and selective in the monitoring the role of P-glycoprotein for the recognition and early treatment of the Alzheimer disease. In addition, up to now none of the studies on interactions of this pump with known inhibitors, report crystallographic data of P-gp inhibitors complexes. Therefore speculating the binding conformation and pose for MC70 might be an added value to a better understanding of the mechanism of action of efflux pumps involved in the Alzheimer's disease.

A view of the refined crystal structure is shown in Figure 1. The packing of the obtained crystal structure is represented in Figure 2; it is interesting observing that the network of the structure features three hydrogen bonds (Table 1): the first between the 2 molecules of methanol, the second between one methanol molecule and the phenolic residue of the molecule and the last between the other methanol molecule and the isoquinoline nucleus. In the crystal weak C—H···O hydrogen bonds also occur. In addition, the pendant biphenyl has an equatorial configuration as proved by a dihedral angle among atoms C8—N1—C7—C18 of -175°.

### S2. Experimental

MC70, [4'-(6,7-dimethoxy-3,4-dihydro-1*H*-isoquinolin-2-yl-methyl)-biphenyl-4-ol] (C_24_H_25_NO_3_) has been obtained after crystallization as yellow needles. The solvent/non-solvent diffusion has been used as crystallization technique: after solubilizing MC70 (5 mg) in methanol (solvent, 1 ml), an equal volume of CH_2_Cl_2_ (non-solvent, 1 ml) has been deposited. The vial has been covered with a perforated cap and left at room temperature. After a couple of days, yellow needles of MC70 2CH_3_OH have grown on the interphase solvent/non-solvent.

### S3. Refinement

The hydrogen atoms of the hydroxyl groups were located by difference Fourier synthesis and freely isotropically refined. The C-bonded H atoms were positioned geometrically with C—H = 0.96, 0.97 and 0.93 Å for methyl, methylene and aromatic H atoms, respectively, and constrained to ride on their parent atoms. The constraint *U*_iso_(H) = *kU*_eq_(C), where *k* = 1.5 for methyl and *k* = 1.2 for aromatic and methylene H atoms, was applied. The highest residual electron density was found 1.59 Å from C16 and the deepest hole 1.04 Å from H12A.

### Crystal data

**Table:**

C_24_H_25_NO_3_·2CH_4_O	*F*(000) = 472
*M**_r_* = 439.53	*D*_x_ = 1.205 Mg m^−^^3^
Monoclinic, *P*2_1_	Mo *K*α radiation, λ = 0.71073 Å
*a* = 8.894 (2) Å	Cell parameters from 130 reflections
*b* = 13.7187 (16) Å	θ = 2.9–26.6°
*c* = 10.680 (2) Å	µ = 0.08 mm^−^^1^
β = 111.575 (17)°	*T* = 293 K
*V* = 1211.8 (4) Å^3^	Needle, yellow
*Z* = 2	0.30 × 0.30 × 0.15 mm

### Data collection

**Table:**

Bruker–Nonius KappaCCD diffractometer	5436 independent reflections
Radiation source: fine-focus sealed tube	2610 reflections with *I* > 2σ(*I*)
Detector resolution: 9.091 pixels mm^-1^	*R*_int_ = 0.116
φ scans and ω scans	θ_max_ = 27.5°, θ_min_ = 5.1°
Absorption correction: multi-scan (*SADABS*; Sheldrick, 2008a)	*h* = −11→11
*T*_min_ = 0.921, *T*_max_ = 0.988	*k* = −16→17
14813 measured reflections	*l* = −13→13

### Refinement

**Table:**

Refinement on *F*^2^	Primary atom site location: structure-invariant direct methods
Least-squares matrix: full	Secondary atom site location: difference Fourier map
*R*[*F*^2^ > 2σ(*F*^2^)] = 0.061	Hydrogen site location: mixed
*wR*(*F*^2^) = 0.115	H atoms treated by a mixture of independent and constrained refinement
*S* = 0.96	*w* = 1/[σ^2^(*F*_o_^2^) + (0.0345*P*)^2^] where *P* = (*F*_o_^2^ + 2*F*_c_^2^)/3
5436 reflections	(Δ/σ)_max_ < 0.001
305 parameters	Δρ_max_ = 0.17 e Å^−^^3^
1 restraint	Δρ_min_ = −0.15 e Å^−^^3^

### Special details

**Table:**

Geometry. All e.s.d.'s (except the e.s.d. in the dihedral angle between two l.s. planes) are estimated using the full covariance matrix. The cell e.s.d.'s are taken into account individually in the estimation of e.s.d.'s in distances, angles and torsion angles; correlations between e.s.d.'s in cell parameters are only used when they are defined by crystal symmetry. An approximate (isotropic) treatment of cell e.s.d.'s is used for estimating e.s.d.'s involving l.s. planes.
Refinement. Refinement of *F*^2^ against ALL reflections. The weighted *R*-factor *wR* and goodness of fit *S* are based on *F*^2^, conventional *R*-factors *R* are based on *F*, with *F* set to zero for negative *F*^2^. The threshold expression of *F*^2^ > 2σ(*F*^2^) is used only for calculating *R*-factors(gt) *etc*. and is not relevant to the choice of reflections for refinement. *R*-factors based on *F*^2^ are statistically about twice as large as those based on *F*, and *R*-factors based on ALL data will be even larger.

### Fractional atomic coordinates and isotropic or equivalent isotropic displacement parameters (Å^2^)

**Table:**

	*x*	*y*	*z*	*U*_iso_*/*U*_eq_	
O1	0.4571 (4)	0.0371 (2)	0.1278 (3)	0.0510 (9)	
O2	0.2161 (4)	0.4009 (3)	0.3492 (4)	0.0591 (10)	
H2O	0.284 (6)	0.426 (4)	0.307 (5)	0.061 (15)*	
O3	0.1944 (4)	0.0677 (2)	−0.0770 (4)	0.0660 (10)	
O4	−0.0551 (4)	1.1537 (3)	0.4038 (4)	0.0686 (11)	
H4O	−0.058 (7)	1.200 (4)	0.337 (6)	0.09 (2)*	
N1	0.4249 (4)	0.4745 (3)	0.2281 (4)	0.0448 (10)	
C1	0.2616 (5)	0.8125 (3)	0.3304 (4)	0.0390 (10)	
C2	0.1761 (5)	0.9019 (3)	0.3470 (4)	0.0409 (11)	
C3	0.4907 (6)	0.2102 (4)	0.1807 (5)	0.0464 (12)	
H3	0.5871	0.1998	0.2530	0.056*	
C4	0.4297 (5)	0.3056 (4)	0.1500 (5)	0.0450 (12)	
C5	0.4095 (6)	0.1326 (3)	0.1054 (5)	0.0450 (12)	
C6	0.3543 (6)	0.6482 (3)	0.3954 (5)	0.0540 (13)	
H6	0.3556	0.5936	0.4475	0.065*	
C7	0.5309 (5)	0.5553 (3)	0.3000 (5)	0.0532 (14)	
H7A	0.6029	0.5719	0.2535	0.064*	
H7B	0.5969	0.5339	0.3900	0.064*	
O5	−0.0663 (6)	0.3050 (3)	0.2485 (5)	0.0724 (12)	
H5O	0.007 (9)	0.331 (6)	0.259 (8)	0.12 (4)*	
C8	0.5275 (5)	0.3884 (3)	0.2354 (5)	0.0519 (13)	
H8A	0.5766	0.3671	0.3283	0.062*	
H8B	0.6136	0.4061	0.2045	0.062*	
C9	0.2058 (6)	0.2421 (3)	−0.0333 (5)	0.0510 (13)	
H9	0.1102	0.2529	−0.1063	0.061*	
C10	0.0835 (6)	0.9047 (4)	0.4265 (5)	0.0532 (13)	
H10	0.0709	0.8477	0.4688	0.064*	
C11	0.2866 (5)	0.3213 (3)	0.0452 (5)	0.0456 (12)	
C12	0.2151 (6)	0.4222 (3)	0.0158 (5)	0.0514 (13)	
H12A	0.1242	0.4268	0.0452	0.062*	
H12B	0.1750	0.4335	−0.0806	0.062*	
C13	0.3421 (6)	0.8087 (3)	0.2410 (4)	0.0491 (12)	
H13	0.3378	0.8621	0.1862	0.059*	
C14	0.1866 (6)	0.9887 (3)	0.2849 (6)	0.0602 (15)	
H14	0.2464	0.9906	0.2295	0.072*	
C15	0.3377 (6)	0.5000 (3)	0.0859 (5)	0.0527 (14)	
H15A	0.4142	0.5069	0.0411	0.063*	
H15B	0.2831	0.5619	0.0803	0.063*	
C16	0.2641 (6)	0.1490 (4)	−0.0050 (5)	0.0469 (11)	
C17	0.2690 (6)	0.7294 (3)	0.4065 (5)	0.0542 (14)	
H17	0.2151	0.7285	0.4664	0.065*	
C18	0.4376 (5)	0.6453 (3)	0.3097 (5)	0.0459 (12)	
C19	0.0090 (6)	0.9887 (4)	0.4456 (5)	0.0578 (14)	
H19	−0.0502	0.9877	0.5015	0.069*	
C20	0.0220 (6)	1.0731 (4)	0.3825 (5)	0.0522 (13)	
C21	0.4286 (6)	0.7268 (3)	0.2323 (5)	0.0538 (13)	
H21	0.4823	0.7270	0.1723	0.065*	
C22	0.1113 (7)	1.0729 (4)	0.3026 (6)	0.0646 (15)	
H22	0.1216	1.1300	0.2594	0.078*	
C23	0.0516 (7)	0.0832 (5)	−0.1916 (6)	0.0864 (19)	
H23A	0.0125	0.0219	−0.2346	0.130*	
H23B	0.0750	0.1257	−0.2535	0.130*	
H23C	−0.0293	0.1126	−0.1641	0.130*	
C24	0.3132 (7)	0.3394 (4)	0.4543 (6)	0.0847 (19)	
H24A	0.2517	0.3176	0.5063	0.127*	
H24B	0.4062	0.3749	0.5112	0.127*	
H24C	0.3475	0.2841	0.4165	0.127*	
C25	0.6072 (7)	0.0187 (4)	0.2328 (6)	0.0707 (17)	
H25A	0.6324	−0.0494	0.2345	0.106*	
H25B	0.6006	0.0371	0.3174	0.106*	
H25C	0.6903	0.0561	0.2179	0.106*	
C26	−0.1413 (7)	0.2993 (5)	0.1057 (6)	0.0846 (19)	
H26A	−0.1724	0.3634	0.0694	0.127*	
H26B	−0.2353	0.2585	0.0821	0.127*	
H26C	−0.0665	0.2721	0.0694	0.127*	

### Atomic displacement parameters (Å^2^)

**Table:**

	*U*^11^	*U*^22^	*U*^33^	*U*^12^	*U*^13^	*U*^23^
O1	0.052 (2)	0.045 (2)	0.058 (2)	0.0013 (16)	0.0214 (19)	−0.0015 (16)
O2	0.045 (2)	0.069 (2)	0.061 (2)	0.0081 (19)	0.0175 (19)	0.016 (2)
O3	0.060 (2)	0.054 (2)	0.067 (3)	−0.0115 (19)	0.004 (2)	−0.016 (2)
O4	0.079 (3)	0.058 (2)	0.085 (3)	0.008 (2)	0.049 (2)	−0.005 (3)
N1	0.039 (2)	0.045 (2)	0.045 (3)	−0.0019 (19)	0.0086 (18)	−0.0088 (19)
C1	0.037 (3)	0.045 (3)	0.034 (3)	−0.009 (2)	0.012 (2)	−0.009 (2)
C2	0.039 (3)	0.047 (3)	0.035 (3)	−0.006 (2)	0.012 (2)	−0.004 (2)
C3	0.042 (3)	0.058 (3)	0.039 (3)	0.004 (3)	0.014 (2)	−0.008 (3)
C4	0.039 (3)	0.051 (3)	0.047 (3)	−0.002 (3)	0.019 (2)	−0.013 (3)
C5	0.047 (3)	0.044 (3)	0.049 (3)	−0.001 (2)	0.023 (3)	−0.005 (2)
C6	0.067 (3)	0.040 (3)	0.055 (3)	−0.004 (3)	0.023 (3)	0.003 (3)
C7	0.049 (3)	0.048 (3)	0.062 (3)	−0.004 (3)	0.020 (3)	−0.015 (3)
O5	0.071 (3)	0.067 (3)	0.075 (3)	−0.010 (3)	0.022 (2)	0.001 (2)
C8	0.042 (3)	0.047 (3)	0.065 (4)	0.003 (2)	0.017 (3)	−0.013 (3)
C9	0.044 (3)	0.054 (3)	0.047 (3)	−0.002 (3)	0.007 (2)	−0.007 (3)
C10	0.061 (3)	0.056 (3)	0.051 (3)	0.002 (3)	0.030 (3)	0.011 (3)
C11	0.043 (3)	0.045 (3)	0.047 (3)	0.002 (2)	0.014 (3)	−0.009 (2)
C12	0.047 (3)	0.050 (3)	0.049 (3)	0.004 (3)	0.009 (2)	−0.003 (3)
C13	0.058 (3)	0.044 (3)	0.048 (3)	−0.003 (3)	0.021 (3)	−0.001 (2)
C14	0.070 (4)	0.049 (3)	0.081 (4)	0.005 (3)	0.052 (3)	0.004 (3)
C15	0.054 (3)	0.052 (3)	0.053 (4)	−0.004 (3)	0.020 (3)	−0.004 (3)
C16	0.047 (3)	0.048 (3)	0.047 (3)	−0.007 (3)	0.018 (3)	−0.010 (3)
C17	0.070 (3)	0.047 (3)	0.057 (4)	−0.007 (3)	0.036 (3)	−0.004 (3)
C18	0.043 (3)	0.045 (3)	0.046 (3)	−0.004 (2)	0.012 (2)	−0.009 (3)
C19	0.062 (3)	0.062 (4)	0.063 (4)	0.005 (3)	0.039 (3)	0.001 (3)
C20	0.049 (3)	0.049 (3)	0.063 (3)	−0.003 (3)	0.026 (3)	−0.007 (3)
C21	0.065 (3)	0.047 (3)	0.057 (4)	−0.008 (3)	0.032 (3)	−0.008 (3)
C22	0.074 (4)	0.052 (3)	0.089 (4)	−0.002 (3)	0.055 (4)	0.007 (3)
C23	0.074 (4)	0.078 (4)	0.079 (4)	−0.017 (3)	−0.005 (4)	−0.024 (4)
C24	0.074 (4)	0.100 (5)	0.067 (4)	0.020 (4)	0.010 (3)	0.028 (4)
C25	0.069 (4)	0.063 (4)	0.068 (4)	0.015 (3)	0.012 (3)	0.002 (3)
C26	0.078 (4)	0.106 (5)	0.073 (5)	−0.022 (4)	0.031 (4)	−0.018 (4)

### Geometric parameters (Å, º)

**Table:**

O1—C5	1.371 (5)	C9—C11	1.399 (6)
O1—C25	1.415 (6)	C9—H9	0.9300
O2—C24	1.415 (6)	C10—C19	1.380 (7)
O2—H2O	0.94 (5)	C10—H10	0.9300
O3—C16	1.367 (6)	C11—C12	1.508 (6)
O3—C23	1.419 (6)	C12—C15	1.513 (6)
O4—C20	1.364 (5)	C12—H12A	0.9700
O4—H4O	0.95 (6)	C12—H12B	0.9700
N1—C15	1.471 (6)	C13—C21	1.383 (6)
N1—C7	1.475 (5)	C13—H13	0.9300
N1—C8	1.477 (5)	C14—C22	1.382 (7)
C1—C17	1.388 (6)	C14—H14	0.9300
C1—C13	1.389 (6)	C15—H15A	0.9700
C1—C2	1.488 (6)	C15—H15B	0.9700
C2—C14	1.383 (6)	C17—H17	0.9300
C2—C10	1.384 (6)	C18—C21	1.376 (6)
C3—C5	1.369 (6)	C19—C20	1.366 (6)
C3—C4	1.409 (6)	C19—H19	0.9300
C3—H3	0.9300	C20—C22	1.364 (6)
C4—C11	1.367 (6)	C21—H21	0.9300
C4—C8	1.516 (6)	C22—H22	0.9300
C5—C16	1.411 (6)	C23—H23A	0.9600
C6—C18	1.373 (6)	C23—H23B	0.9600
C6—C17	1.378 (6)	C23—H23C	0.9600
C6—H6	0.9300	C24—H24A	0.9600
C7—C18	1.511 (6)	C24—H24B	0.9600
C7—H7A	0.9700	C24—H24C	0.9600
C7—H7B	0.9700	C25—H25A	0.9600
O5—C26	1.424 (7)	C25—H25B	0.9600
O5—H5O	0.71 (8)	C25—H25C	0.9600
C8—H8A	0.9700	C26—H26A	0.9600
C8—H8B	0.9700	C26—H26B	0.9600
C9—C16	1.370 (7)	C26—H26C	0.9600
			
C5—O1—C25	116.7 (4)	C1—C13—H13	119.4
C24—O2—H2O	107 (3)	C22—C14—C2	122.1 (5)
C16—O3—C23	116.1 (4)	C22—C14—H14	118.9
C20—O4—H4O	107 (3)	C2—C14—H14	118.9
C15—N1—C7	110.6 (4)	N1—C15—C12	110.7 (4)
C15—N1—C8	109.0 (4)	N1—C15—H15A	109.5
C7—N1—C8	108.0 (3)	C12—C15—H15A	109.5
C17—C1—C13	116.5 (4)	N1—C15—H15B	109.5
C17—C1—C2	121.6 (4)	C12—C15—H15B	109.5
C13—C1—C2	121.9 (4)	H15A—C15—H15B	108.1
C14—C2—C10	115.5 (4)	O3—C16—C9	125.5 (4)
C14—C2—C1	121.6 (4)	O3—C16—C5	115.2 (5)
C10—C2—C1	122.8 (4)	C9—C16—C5	119.3 (5)
C5—C3—C4	120.7 (4)	C6—C17—C1	121.5 (5)
C5—C3—H3	119.6	C6—C17—H17	119.2
C4—C3—H3	119.6	C1—C17—H17	119.2
C11—C4—C3	120.0 (4)	C6—C18—C21	116.7 (5)
C11—C4—C8	122.0 (4)	C6—C18—C7	120.9 (5)
C3—C4—C8	118.0 (4)	C21—C18—C7	122.3 (5)
C3—C5—O1	125.4 (5)	C20—C19—C10	120.2 (5)
C3—C5—C16	119.3 (5)	C20—C19—H19	119.9
O1—C5—C16	115.3 (4)	C10—C19—H19	119.9
C18—C6—C17	122.0 (5)	C22—C20—O4	123.4 (5)
C18—C6—H6	119.0	C22—C20—C19	118.8 (5)
C17—C6—H6	119.0	O4—C20—C19	117.8 (5)
N1—C7—C18	112.8 (4)	C18—C21—C13	122.1 (5)
N1—C7—H7A	109.0	C18—C21—H21	119.0
C18—C7—H7A	109.0	C13—C21—H21	119.0
N1—C7—H7B	109.0	C20—C22—C14	120.7 (5)
C18—C7—H7B	109.0	C20—C22—H22	119.6
H7A—C7—H7B	107.8	C14—C22—H22	119.6
C26—O5—H5O	104 (7)	O3—C23—H23A	109.5
N1—C8—C4	111.2 (4)	O3—C23—H23B	109.5
N1—C8—H8A	109.4	H23A—C23—H23B	109.5
C4—C8—H8A	109.4	O3—C23—H23C	109.5
N1—C8—H8B	109.4	H23A—C23—H23C	109.5
C4—C8—H8B	109.4	H23B—C23—H23C	109.5
H8A—C8—H8B	108.0	O2—C24—H24A	109.5
C16—C9—C11	121.5 (5)	O2—C24—H24B	109.5
C16—C9—H9	119.3	H24A—C24—H24B	109.5
C11—C9—H9	119.3	O2—C24—H24C	109.5
C19—C10—C2	122.7 (5)	H24A—C24—H24C	109.5
C19—C10—H10	118.7	H24B—C24—H24C	109.5
C2—C10—H10	118.7	O1—C25—H25A	109.5
C4—C11—C9	119.1 (4)	O1—C25—H25B	109.5
C4—C11—C12	120.6 (4)	H25A—C25—H25B	109.5
C9—C11—C12	120.2 (4)	O1—C25—H25C	109.5
C11—C12—C15	111.8 (4)	H25A—C25—H25C	109.5
C11—C12—H12A	109.2	H25B—C25—H25C	109.5
C15—C12—H12A	109.2	O5—C26—H26A	109.5
C11—C12—H12B	109.2	O5—C26—H26B	109.5
C15—C12—H12B	109.2	H26A—C26—H26B	109.5
H12A—C12—H12B	107.9	O5—C26—H26C	109.5
C21—C13—C1	121.1 (4)	H26A—C26—H26C	109.5
C21—C13—H13	119.4	H26B—C26—H26C	109.5
			
C17—C1—C2—C14	171.2 (5)	C1—C2—C14—C22	−178.2 (5)
C13—C1—C2—C14	−6.9 (6)	C7—N1—C15—C12	−173.6 (4)
C17—C1—C2—C10	−7.7 (7)	C8—N1—C15—C12	67.8 (5)
C13—C1—C2—C10	174.2 (5)	C11—C12—C15—N1	−47.5 (5)
C5—C3—C4—C11	1.1 (6)	C23—O3—C16—C9	1.4 (7)
C5—C3—C4—C8	−178.6 (4)	C23—O3—C16—C5	−177.6 (4)
C4—C3—C5—O1	−179.1 (4)	C11—C9—C16—O3	−179.2 (4)
C4—C3—C5—C16	0.7 (6)	C11—C9—C16—C5	−0.3 (7)
C25—O1—C5—C3	−3.8 (6)	C3—C5—C16—O3	177.9 (4)
C25—O1—C5—C16	176.4 (4)	O1—C5—C16—O3	−2.3 (5)
C15—N1—C7—C18	65.8 (5)	C3—C5—C16—C9	−1.1 (6)
C8—N1—C7—C18	−175.0 (4)	O1—C5—C16—C9	178.7 (4)
C15—N1—C8—C4	−53.1 (5)	C18—C6—C17—C1	0.6 (7)
C7—N1—C8—C4	−173.4 (4)	C13—C1—C17—C6	1.1 (7)
C11—C4—C8—N1	22.3 (6)	C2—C1—C17—C6	−177.0 (4)
C3—C4—C8—N1	−158.0 (4)	C17—C6—C18—C21	−1.6 (7)
C14—C2—C10—C19	−1.4 (7)	C17—C6—C18—C7	179.7 (4)
C1—C2—C10—C19	177.6 (5)	N1—C7—C18—C6	73.2 (6)
C3—C4—C11—C9	−2.4 (6)	N1—C7—C18—C21	−105.4 (5)
C8—C4—C11—C9	177.3 (4)	C2—C10—C19—C20	1.5 (8)
C3—C4—C11—C12	176.6 (4)	C10—C19—C20—C22	−0.9 (8)
C8—C4—C11—C12	−3.8 (6)	C10—C19—C20—O4	179.4 (5)
C16—C9—C11—C4	2.0 (7)	C6—C18—C21—C13	0.9 (7)
C16—C9—C11—C12	−176.9 (4)	C7—C18—C21—C13	179.6 (4)
C4—C11—C12—C15	15.9 (6)	C1—C13—C21—C18	0.8 (8)
C9—C11—C12—C15	−165.2 (4)	O4—C20—C22—C14	180.0 (5)
C17—C1—C13—C21	−1.8 (7)	C19—C20—C22—C14	0.4 (8)
C2—C1—C13—C21	176.3 (4)	C2—C14—C22—C20	−0.3 (9)
C10—C2—C14—C22	0.8 (8)		

### Hydrogen-bond geometry (Å, º)

**Table:**

*D*—H···*A*	*D*—H	H···*A*	*D*···*A*	*D*—H···*A*
O2—H2*O*···N1	0.94 (5)	1.87 (5)	2.812 (5)	178 (4)
O4—H4*O*···O5^i^	0.95 (6)	1.71 (6)	2.636 (6)	165 (5)
O5—H5*O*···O2	0.71 (8)	2.00 (8)	2.684 (6)	162 (9)
C15—H15*A*···O1^ii^	0.97	2.50	3.445 (6)	164
C23—H23*A*···O2^iii^	0.96	2.56	3.437 (6)	152

Symmetry codes: (i) *x*, *y*+1, *z*; (ii) −*x*+1, *y*+1/2, −*z*; (iii) −*x*, *y*−1/2, −*z*.
