# Supplementary material for: Crystallographic study of PET radio­tracers in clinical evaluation for early diagnosis of Alzheimers
Source: Acta Crystallogr Sect E Struct Rep Online. 2014 Oct 4;70(Pt 11):o1149–50. doi: 10.1107/S1600536814021400 (PMC4257311; doi:10.1107/S1600536814021400)
Supplement: Supplementary file 3 [file e-70-o1149-Isup3.docx]

**Supplementary materials**

**Crystallographic study of PET radiotracers in clinical evaluation for early diagnosis of Alzheimer**

**Angela Altomare, Elena Capparelli, Antonio Carrieri, Nicola A. Colabufo, Anna Moliterni, Rosanna Rizzi, Dritan Siliqi**

**4’-(6,7-dimethoxy-3,4-dihydro-1*H*-isoquinolin-2-yl-methyl)-biphenyl-4-ol (MC70)**

| *Crystal data* |  |
| --- | --- |
| C_24_H_25_NO_3_·2CH_3_OH | *F*(000) = 472 |
| *M_r_ =* 439.53 | *D_x_* = 1.205 Mg m^-3^ |
| Monoclinic, *P*2_1_ | Mo K_α_ radiation, λ = 0.71073 Å |
| Hall symbol: P 2yb | Cell parameters from 130 reflections |
| *a* = 8.894 (2) Å | *θ* = 5.1-27.5^o^ |
| *b* = 13.7187 (16) Å | *μ* = 0.083 mm^-1^ |
| *c* = 10.680 (2) Å | *T* = 293 K |
| *β =* 111.575 (17)^o^ | Needle, yellow |
| *V* = 1211.8 (4) Å^3^ | 0.3 × 0.3 × 0.15 mm |
| *Z* = 2 |  |
|  |  |
| *Data collection* |  |
| Bruker-Nonius KappaCCD area | 14813 measured reflections |
| detector diffractometer | 5436 independent reflections |
| Radiation source: fine-focus sealed tube | 2610 reflections with *I* > 2σ(*I*) |
| Graphite monochromator | R_int_ = 0.1160 |
| Detector resolution: 9.091 pixels mm^-1^ | *θ*_max_ = 27.5^o^, *θ*_min_ = 5.1^o^ |
| ϕ and ω scans | *h* = -11→11 |
| Absorption correction: multi-scan | *k* = -16→17 |
| (*SADABS*; Sheldrick, 2008a) | *l* = -13→13 |
| T_min_ = 0.921, T_max_ = 0.988 |  |
|  |  |
| \| *Refinement* \| \| --- \| |  |
| Refinement on *F*^2^ | Hydrogen site location: a mixture of inferred |
| Least-squares matrix: full | from neighbouring sites and difference Fourier |
| *R*[*F*^2^>2σ(*F*^2^)] = 0.0607 | location |
| *wR(F*^2^) = 0.1147 | H atoms refinement: a mixture of independent |
| S = 0.964 | and constrained refinement |
| 5436 reflections | $w=1/\left[\sigma^{2}\left(\text{F}_{o}^{2}\right)+\left(0.0345\text{P}\right)^{2}\right]$ |
| 305 parameters | where $\text{P}=\left(\text{F}_{o}^{2}+2\text{F}_{c}^{2}\right)/3$ |
| 1 restraints | (Δ/σ)_max_ < 0.001 |
| Primary atom site location: structure invariant | Δρ_max_ = 0.171 e Å^-3^ |
| direct methods | Δρ_min_ = -0.151 e Å^-3^ |
| Secondary atom site location: Difference |  |
| Fourier map |  |

*Special details*

**Geometry**. All e.s.d.’s (except the e.s.d. in the dihedral angle between two l.s. planes) are estimated using the full covariance matrix. The cell e.s.d.’s are taken into account individually in the estimation of e.s.d.’s in distances, angles and torsion angles; correlations between e.s.d.’s in cell parameters are only used when they are defined by crystal symmetry. An approximate (isotropic) treatment of cell e.s.d.’s is used for estimating e.s.d.’s involving l.s. planes.

**Refinement**. Refinement of F^2^ against ALL reflections. The weighted R-factor wR and goodness of fit S are based on F^2^, conventional R-factors R are based on F, with F set to zero for negative F^2^ . The threshold expression of F^2^ > 2σ(F^2^) is used only for calculating R-factors(gt) etc. and is not relevant to the choice of reflections for refinement. R-factors based on F^2^ are statistically about twice as large as those based on F, and R-factors based on ALL data will be even larger.

*Fractional atomic coordinates and isotropic or equivalent isotropic displacement parameters* (*Å^2^*)

|  | *x* | *y* | *z* | *U*_iso_^*^/*U*_eq_ |
| --- | --- | --- | --- | --- |
| O1 | 0.4571(4) | 0.0371(2) | 0.1278(3) | 0.0510(9) |
| O2 | 0.2161(4) | 0.4009(3) | 0.3492(4) | 0.0591(10) |
| H2O | 0.284(6) | 0.426(4) | 0.307(5) | 0.061(15)^*^ |
| O3 | 0.1944(4) | 0.0677(2) | -0.0770(4) | 0.0660(10) |
| O4 | -0.0551(4) | 1.1537(3) | 0.4038(4) | 0.0686(11) |
| H4O | -0.058(7) | 1.200(4) | 0.337(6) | 0.09(2)^*^ |
| N1 | 0.4249(4) | 0.4745(3) | 0.2281(4) | 0.0448(10) |
| C1 | 0.2616(5) | 0.8125(3) | 0.3304(4) | 0.0390(10) |
| C2 | 0.1761(5) | 0.9019(3) | 0.3470(4) | 0.0409(11) |
| C3 | 0.4907(6) | 0.2102(4) | 0.1807(5) | 0.0464(12) |
| H3 | 0.5871 | 0.1998 | 0.2530 | 0.056^*^ |
| C4 | 0.4297(5) | 0.3056(4) | 0.1500(5) | 0.0450(12) |
| C5 | 0.4095(6) | 0.1326(3) | 0.1054(5) | 0.0450(12) |
| C6 | 0.3543(6) | 0.6482(3) | 0.3954(5) | 0.0540(13) |
| H6 | 0.3556 | 0.5936 | 0.4475 | 0.065^*^ |
| C7 | 0.5309(5) | 0.5553(3) | 0.3000(5) | 0.0532(14) |
| H7A | 0.6029 | 0.5719 | 0.2535 | 0.064^*^ |
| H7B | 0.5969 | 0.5339 | 0.3900 | 0.064^*^ |
| O5 | -0.0663(6) | 0.3050(3) | 0.2485(5) | 0.0724(12) |
| H5O | 0.007(9) | 0.331(6) | 0.259(8) | 0.12(4)^*^ |
| C8 | 0.5275(5) | 0.3884(3) | 0.2354(5) | 0.0519(13) |
| H8A | 0.5766 | 0.3671 | 0.3283 | 0.062^*^ |
| H8B | 0.6136 | 0.4061 | 0.2045 | 0.062^*^ |
| C9 | 0.2058(6) | 0.2421(3) | -0.0333(5) | 0.0510(13) |
| H9 | 0.1102 | 0.2529 | -0.1063 | 0.061^*^ |
| C10 | 0.0835(6) | 0.9047(4) | 0.4265(5) | 0.0532(13) |
| H10 | 0.0709 | 0.8477 | 0.4688 | 0.064^*^ |
| C11 | 0.2866(5) | 0.3213(3) | 0.0452(5) | 0.0456(12) |
| C12 | 0.2151(6) | 0.4222(3) | 0.0158(5) | 0.0514(13) |
| H12A | 0.1242 | 0.4268 | 0.0452 | 0.062^*^ |
| H12B | 0.1750 | 0.4335 | -0.0806 | 0.062^*^ |
| C13 | 0.3421(6) | 0.8087(3) | 0.2410(4) | 0.0491(12) |
| H13 | 0.3378 | 0.8621 | 0.1862 | 0.059^*^ |
| C14 | 0.1866(6) | 0.9887(3) | 0.2849(6) | 0.0602(15) |
| H14 | 0.2464 | 0.9906 | 0.2295 | 0.072^*^ |
| C15 | 0.3377(6) | 0.5000(3) | 0.0859(5) | 0.0527(14) |
| H15A | 0.4142 | 0.5069 | 0.0411 | 0.063^*^ |
| H15B | 0.2831 | 0.5619 | 0.0803 | 0.063^*^ |
| C16 | 0.2641(6) | 0.1490(4) | -0.0050(5) | 0.0469(11) |
| C17 | 0.2690(6) | 0.7294(3) | 0.4065(5) | 0.0542(14) |
| H17 | 0.2151 | 0.7285 | 0.4664 | 0.065^*^ |
| C18 | 0.4376(5) | 0.6453(3) | 0.3097(5) | 0.0459(12) |
| C19 | 0.0090(6) | 0.9887(4) | 0.4456(5) | 0.0578(14) |
| H19 | -0.0502 | 0.9877 | 0.5015 | 0.069^*^ |
| C20 | 0.0220(6) | 1.0731(4) | 0.3825(5) | 0.0522(13) |
| C21 | 0.4286(6) | 0.7268(3) | 0.2323(5) | 0.0538(13) |
| H21 | 0.4823 | 0.7270 | 0.1723 | 0.065^*^ |
| C22 | 0.1113(7) | 1.0729(4) | 0.3026(6) | 0.0646(15) |
| H22 | 0.1216 | 1.1300 | 0.2594 | 0.078^*^ |
| C23 | 0.0516(7) | 0.0832(5) | -0.1916(6) | 0.0864(19) |
| H23A | 0.0125 | 0.0219 | -0.2346 | 0.130^*^ |
| H23B | 0.0750 | 0.1257 | -0.2535 | 0.130^*^ |
| H23C | -0.0293 | 0.1126 | -0.1641 | 0.130^*^ |
| C24 | 0.3132(7) | 0.3394(4) | 0.4543(6) | 0.0847(19) |
| H24A | 0.2517 | 0.3176 | 0.5063 | 0.127^*^ |
| H24B | 0.4062 | 0.3749 | 0.5112 | 0.127^*^ |
| H24C | 0.3475 | 0.2841 | 0.4165 | 0.127^*^ |
| C25 | 0.6072(7) | 0.0187(4) | 0.2328(6) | 0.0707(17) |
| H25A | 0.6324 | -0.0494 | 0.2345 | 0.106^*^ |
| H25B | 0.6006 | 0.0371 | 0.3174 | 0.106^*^ |
| H25C | 0.6903 | 0.0561 | 0.2179 | 0.106^*^ |
| C26 | -0.1413(7) | 0.2993(5) | 0.1057(6) | 0.0846(19) |
| H26A | -0.1724 | 0.3634 | 0.0694 | 0.127^*^ |
| H26B | -0.2353 | 0.2585 | 0.0821 | 0.127^*^ |
| H26C | -0.0665 | 0.2721 | 0.0694 | 0.127^*^ |

*Atomic displacement parameters (Å^2^)*

|  | *U*^11^ | *U*^22^ | *U*^33^ | *U*^12^ | *U*^13^ | *U*^23^ |
| --- | --- | --- | --- | --- | --- | --- |
| O1 | 0.052(2) | 0.045(2) | 0.058(2) | 0.0013(16) | 0.0214(19) | -0.0015(16) |
| O2 | 0.045(2) | 0.069(2) | 0.061(2) | 0.0081(19) | 0.0175(19) | 0.016(2) |
| O3 | 0.060(2) | 0.054(2) | 0.067(3) | -0.0115(19) | 0.004(2) | -0.016(2) |
| O4 | 0.079(3) | 0.058(2) | 0.085(3) | 0.008(2) | 0.049(2) | -0.005(3) |
| N1 | 0.039(2) | 0.045(2) | 0.045(3) | -0.0019(19) | 0.0086(18) | -0.0088(19) |
| C1 | 0.037(3) | 0.045(3) | 0.034(3) | -0.009(2) | 0.012(2) | -0.009(2) |
| C2 | 0.039(3) | 0.047(3) | 0.035(3) | -0.006(2) | 0.012(2) | -0.004(2) |
| C3 | 0.042(3) | 0.058(3) | 0.039(3) | 0.004(3) | 0.014(2) | -0.008(3) |
| C4 | 0.039(3) | 0.051(3) | 0.047(3) | -0.002(3) | 0.019(2) | -0.013(3) |
| C5 | 0.047(3) | 0.044(3) | 0.049(3) | -0.001(2) | 0.023(3) | -0.005(2) |
| C6 | 0.067(3) | 0.040(3) | 0.055(3) | -0.004(3) | 0.023(3) | 0.003(3) |
| C7 | 0.049(3) | 0.048(3) | 0.062(3) | -0.004(3) | 0.020(3) | -0.015(3) |
| O5 | 0.071(3) | 0.067(3) | 0.075(3) | -0.010(3) | 0.022(2) | 0.001(2) |
| C8 | 0.042(3) | 0.047(3) | 0.065(4) | 0.003(2) | 0.017(3) | -0.013(3) |
| C9 | 0.044(3) | 0.054(3) | 0.047(3) | -0.002(3) | 0.007(2) | -0.007(3) |
| C10 | 0.061(3) | 0.056(3) | 0.051(3) | 0.002(3) | 0.030(3) | 0.011(3) |
| C11 | 0.043(3) | 0.045(3) | 0.047(3) | 0.002(2) | 0.014(3) | -0.009(2) |
| C12 | 0.047(3) | 0.050(3) | 0.049(3) | 0.004(3) | 0.009(2) | -0.003(3) |
| C13 | 0.058(3) | 0.044(3) | 0.048(3) | -0.003(3) | 0.021(3) | -0.001(2) |
| C14 | 0.070(4) | 0.049(3) | 0.081(4) | 0.005(3) | 0.052(3) | 0.004(3) |
| C15 | 0.054(3) | 0.052(3) | 0.053(4) | -0.004(3) | 0.020(3) | -0.004(3) |
| C16 | 0.047(3) | 0.048(3) | 0.047(3) | -0.007(3) | 0.018(3) | -0.010(3) |
| C17 | 0.070(3) | 0.047(3) | 0.057(4) | -0.007(3) | 0.036(3) | -0.004(3) |
| C18 | 0.043(3) | 0.045(3) | 0.046(3) | -0.004(2) | 0.012(2) | -0.009(3) |
| C19 | 0.062(3) | 0.062(4) | 0.063(4) | 0.005(3) | 0.039(3) | 0.001(3) |
| C20 | 0.049(3) | 0.049(3) | 0.063(3) | -0.003(3) | 0.026(3) | -0.007(3) |
| C21 | 0.065(3) | 0.047(3) | 0.057(4) | -0.008(3) | 0.032(3) | -0.008(3) |
| C22 | 0.074(4) | 0.052(3) | 0.089(4) | -0.002(3) | 0.055(4) | 0.007(3) |
| C23 | 0.074(4) | 0.078(4) | 0.079(4) | -0.017(3) | -0.005(4) | -0.024(4) |
| C24 | 0.074(4) | 0.100(5) | 0.067(4) | 0.020(4) | 0.010(3) | 0.028(4) |
| C25 | 0.069(4) | 0.063(4) | 0.068(4) | 0.015(3) | 0.012(3) | 0.002(3) |
| C26 | 0.078(4) | 0.106(5) | 0.073(5) | -0.022(4) | 0.031(4) | -0.018(4) |

*Geometric parameters (Å, º)*

| O1⎯C5 | 1.371(5) | C9⎯C11 | 1.399(6) |
| --- | --- | --- | --- |
| O1⎯C25 | 1.415(6) | C9⎯H9 | 0.9300 |
| O2⎯C24 | 1.415(6) | C10⎯C19 | 1.380(7) |
| O2⎯H2O | 0.94(5) | C10⎯H10 | 0.9300 |
| O3⎯C16 | 1.367(6) | C11⎯C12 | 1.508(6) |
| O3⎯C23 | 1.419(6) | C12⎯C15 | 1.513(6) |
| O4⎯C20 | 1.364(5) | C12⎯H12A | 0.9700 |
| O4⎯H4O | 0.95(6) | C12⎯H12B | 0.9700 |
| N1⎯C15 | 1.471(6) | C13⎯C21 | 1.383(6) |
| N1⎯C7 | 1.475(5) | C13⎯H13 | 0.9300 |
| N1⎯C8 | 1.477(5) | C14⎯C22 | 1.382(7) |
| C1⎯C17 | 1.388(6) | C14⎯H14 | 0.9300 |
| C1⎯C13 | 1.389(6) | C15⎯H15A | 0.9700 |
| C1⎯C2 | 1.488(6) | C15⎯H15B | 0.9700 |
| C2⎯C14 | 1.383(6) | C17⎯H17 | 0.9300 |
| C2⎯C10 | 1.384(6) | C18⎯C21 | 1.376(6) |
| C3⎯C5 | 1.369(6) | C19⎯C20 | 1.366(6) |
| C3⎯C4 | 1.409(6) | C19⎯H19 | 0.9300 |
| C3⎯H3 | 0.9300 | C20⎯C22 | 1.364(6) |
| C4⎯C11 | 1.367(6) | C21⎯H21 | 0.9300 |
| C4⎯C8 | 1.516(6) | C22⎯H22 | 0.9300 |
| C5⎯C16 | 1.411(6) | C23⎯H23A | 0.9600 |
| C6⎯C18 | 1.373(6) | C23⎯H23B | 0.9600 |
| C6⎯C17 | 1.378(6) | C23⎯H23C | 0.9600 |
| C6⎯H6 | 0.9300 | C24⎯H24A | 0.9600 |
| C7⎯C18 | 1.511(6) | C24⎯H24B | 0.9600 |
| C7⎯H7A | 0.9700 | C24⎯H24C | 0.9600 |
| C7⎯H7B | 0.9700 | C25⎯H25A | 0.9600 |
| O5⎯C26 | 1.424(7) | C25⎯H25B | 0.9600 |
| O5⎯H5O | 0.71(8) | C25⎯H25C | 0.9600 |
| C8⎯H8A | 0.9700 | C26⎯H26A | 0.9600 |
| C8⎯H8B | 0.9700 | C26⎯H26B | 0.9600 |
| C9⎯C16 | 1.370(7) | C26⎯H26C | 0.9600 |
|  |  |  |  |
| C5⎯O1⎯C25 | 116.7(4) | C1⎯C13⎯H13 | 119.4 |
| C24⎯O2⎯H2O | 107(3) | C22⎯C14⎯C2 | 122.1(5) |
| C16⎯O3⎯C23 | 116.1(4) | C22⎯C14⎯H14 | 118.9 |
| C20⎯O4⎯H4O | 107(3) | C2⎯C14⎯H14 | 118.9 |
| C15⎯N1⎯C7 | 110.6(4) | N1⎯C15⎯C12 | 110.7(4) |
| C15⎯N1⎯C8 | 109.0(4) | N1⎯C15⎯H15A | 109.5 |
| C7⎯N1⎯C8 | 108.0(3) | C12⎯C15⎯H15A | 109.5 |
| C17⎯C1⎯C13 | 116.5(4) | N1⎯C15⎯H15B | 109.5 |
| C17⎯C1⎯C2 | 121.6(4) | C12⎯C15⎯H15B | 109.5 |
| C13⎯C1⎯C2 | 121.9(4) | H15A⎯C15⎯H15B | 108.1 |
| C14⎯C2⎯C10 | 115.5(4) | O3⎯C16⎯C9 | 125.5(4) |
| C14⎯C2⎯C1 | 121.6(4) | O3⎯C16⎯C5 | 115.2(5) |
| C10⎯C2⎯C1 | 122.8(4) | C9⎯C16⎯C5 | 119.3(5) |
| C5⎯C3⎯C4 | 120.7(4) | C6⎯C17⎯C1 | 121.5(5) |
| C5⎯C3⎯H3 | 119.6 | C6⎯C17⎯H17 | 119.2 |
| C4⎯C3⎯H3 | 119.6 | C1⎯C17⎯H17 | 119.2 |
| C11⎯C4⎯C3 | 120.0(4) | C6⎯C18⎯C21 | 116.7(5) |
| C11⎯C4⎯C8 | 122.0(4) | C6⎯C18⎯C7 | 120.9(5) |
| C3⎯C4⎯C8 | 118.0(4) | C21⎯C18⎯C7 | 122.3(5) |
| C3⎯C5⎯O1 | 125.4(5) | C20⎯C19⎯C10 | 120.2(5) |
| C3⎯C5⎯C16 | 119.3(5) | C20⎯C19⎯H19 | 119.9 |
| O1⎯C5⎯C16 | 115.3(4) | C10⎯C19⎯H19 | 119.9 |
| C18⎯C6⎯C17 | 122.0(5) | C22⎯C20⎯O4 | 123.4(5) |
| C18⎯C6⎯H6 | 119.0 | C22⎯C20⎯C19 | 118.8(5) |
| C17⎯C6⎯H6 | 119.0 | O4⎯C20⎯ C19 | 117.8(5) |
| N1⎯C7⎯C18 | 112.8(4) | C18⎯C21⎯C13 | 122.1(5) |
| N1⎯C7⎯H7A | 109.0 | C18⎯C21⎯H21 | 119.0 |
| C18⎯C7⎯H7A | 109.0 | C13⎯C21⎯H21 | 119.0 |
| N1⎯C7⎯H7B | 109.0 | C20⎯C22⎯C14 | 120.7(5) |
| C18⎯C7⎯H7B | 109.0 | C20⎯C22⎯H22 | 119.6 |
| H7A⎯C7⎯H7B | 107.8 | C14⎯C22⎯H22 | 119.6 |
| C26⎯O5⎯H5O | 104(7) | O3⎯C23⎯H23A | 109.5 |
| N1⎯C8⎯C4 | 111.2(4) | O3⎯C23⎯H23B | 109.5 |
| N1⎯C8⎯H8A | 109.4 | H23A⎯C23⎯H23B | 109.5 |
| C4⎯C8⎯H8A | 109.4 | O3⎯C23⎯H23C | 109.5 |
| N1⎯C8⎯H8B | 109.4 | H23A⎯C23⎯H23C | 109.5 |
| C4⎯C8⎯H8B | 109.4 | H23B⎯C23⎯H23C | 109.5 |
| H8A⎯C8⎯H8B | 108.0 | O2⎯C24⎯H24A | 109.5 |
| C16⎯C9⎯C11 | 121.5(5) | O2⎯C24⎯H24B | 109.5 |
| C16⎯C9⎯H9 | 119.3 | H24A⎯C24⎯H24B | 109.5 |
| C11⎯C9⎯H9 | 119.3 | O2⎯C24⎯H24C | 109.5 |
| C19⎯C10⎯C2 | 122.7(5) | H24A⎯C24⎯H24C | 109.5 |
| C19⎯C10⎯H10 | 118.7 | H24B⎯C24⎯H24C | 109.5 |
| C2⎯C10⎯H10 | 118.7 | O1⎯C25⎯H25A | 109.5 |
| C4⎯C11⎯C9 | 119.1(4) | O1⎯C25⎯H25B | 109.5 |
| C4⎯C11⎯C12 | 120.6(4) | H25A⎯C25⎯H25B | 109.5 |
| C9⎯C11⎯C12 | 120.2(4) | O1⎯C25⎯H25C | 109.5 |
| C11⎯C12⎯C15 | 111.8(4) | H25A⎯C25⎯H25C | 109.5 |
| C11⎯C12⎯H12A | 109.2 | H25B⎯C25⎯H25C | 109.5 |
| C15⎯C12⎯H12A | 109.2 | O5⎯C26⎯H26A | 109.5 |
| C11⎯C12⎯H12B | 109.2 | O5⎯C26⎯H26B | 109.5 |
| C15⎯C12⎯H12B | 109.2 | H26A⎯C26⎯H26B | 109.5 |
| H12A⎯C12⎯H12B | 107.9 | O5⎯C26⎯H26C | 109.5 |
| C21⎯C13⎯C1 | 121.1(4) | H26A⎯C26⎯H26C | 109.5 |
| C21⎯C13⎯H13 | 119.4 | H26B C26 H26C | 109.5 |
|  |  |  |  |
| C17⎯C1⎯C2⎯C14 | 171.2(5) | C1⎯C2⎯C14⎯C22 | -178.2(5) |
| C13⎯C1⎯C2⎯C14 | -6.9(6) | C7⎯N1⎯C15⎯C12 | -173.6(4) |
| C17⎯C1⎯C2⎯C10 | -7.7(7) | C8⎯N1⎯C15⎯C12 | 67.8(5) |
| C13⎯C1⎯C2⎯C10 | 174.2(5) | C11⎯C12⎯C15⎯N1 | -47.5(5) |
| C5⎯C3⎯C4⎯C11 | 1.1(6) | C23⎯O3⎯C16⎯C9 | 1.4(7) |
| C5⎯C3⎯C4⎯C8 | -178.6(4) | C23⎯O3⎯C16⎯C5 | -177.6(4) |
| C4⎯C3⎯C5⎯O1 | -179.1(4) | C11⎯C9⎯C16⎯O3 | -179.2(4) |
| C4⎯C3⎯C5⎯C16 | 0.7(6) | C11⎯C9⎯C16⎯C5 | -0.3(7) |
| C25⎯O1⎯C5⎯C3 | -3.8(6) | C3⎯C5⎯C16⎯O3 | 177.9(4) |
| C25⎯O1⎯C5⎯C16 | 176.4(4) | O1⎯C5⎯C16⎯O3 | -2.3(5) |
| C15⎯N1⎯C7⎯C18 | 65.8(5) | C3⎯C5⎯C16⎯C9 | -1.1(6) |
| C8⎯N1⎯C7⎯C18 | -175.0(4) | O1⎯C5⎯C16⎯C9 | 178.7(4) |
| C15⎯N1⎯C8⎯C4 | -53.1(5) | C18⎯C6⎯C17⎯C1 | 0.6(7) |
| C7⎯N1⎯C8⎯C4 | -173.4(4) | C13⎯C1⎯C17⎯C6 | 1.1(7) |
| C11⎯C4⎯C8⎯N1 | 22.3(6) | C2⎯C1⎯C17⎯C6 | -177.0(4) |
| C3⎯C4⎯C8⎯N1 | -158.0(4) | C17⎯C6⎯C18⎯C21 | -1.6(7) |
| C14⎯C2⎯C10⎯C19 | -1.4(7) | C17⎯C6⎯C18⎯C7 | 179.7(4) |
| C1⎯C2⎯C10⎯C19 | 177.6(5) | N1⎯C7⎯C18⎯C6 | 73.2(6) |
| C3⎯C4⎯C11⎯C9 | -2.4(6) | N1⎯C7⎯C18⎯C21 | -105.4(5) |
| C8⎯C4⎯C11⎯C9 | 177.3(4) | C2⎯C10⎯C19⎯C20 | 1.5(8) |
| C3⎯C4⎯C11⎯C12 | 176.6(4) | C10⎯C19⎯C20⎯C22 | -0.9(8) |
| C8⎯C4⎯C11⎯C12 | -3.8(6) | C10⎯C19⎯C20⎯O4 | 179.4(5) |
| C16⎯C9⎯C11⎯C4 | 2.0(7) | C6⎯C18⎯C21⎯C13 | 0.9(7) |
| C16⎯C9⎯C11⎯C12 | -176.9(4) | C7⎯C18⎯C21⎯C13 | 179.6(4) |
| C4⎯C11⎯C12⎯C15 | 15.9(6) | C1⎯C13⎯C21⎯C18 | 0.8(8) |
| C9⎯C11⎯C12⎯C15 | -165.2(4) | O4⎯C20⎯C22⎯C14 | 180.0(5) |
| C17⎯C1⎯C13⎯C21 | -1.8(7) | C19⎯C20⎯C22⎯C14 | 0.4(8) |
| C2⎯C1⎯C13⎯C21 | 176.3(4) | C2⎯C14⎯C22⎯C20 | -0.3(9) |
| C10⎯C2⎯C14⎯C22 | 0.8(8) |  |  |

*Hydrogen-bond geometry (Å, º)*

| *D* − H···*A* | *D* − H | H···*A* | *D*···*A* | *D* − H···*A* |
| --- | --- | --- | --- | --- |
| O2 − H2O···N1 | 0.94(5) | 1.87(5) | 2.812(5) | 178(4) |
| O4 − H4O···O5^i^ | 0.95(6) | 1.71(6) | 2.636(6) | 165(5) |
| O5 − H5O···O2 | 0.71(8) | 2.00(8) | 2.684(6) | 162(9) |
| C15 − H15*A*···O1^ii^ | 0.97 | 2.50 | 3.445(6) | 164.3 |
| C23 − H23*A*···O2^iii^ | 0.96 | 2.56 | 3.437(6) | 151.8 |

Symmetry codes: (i) *x*, *y*+1, *z*; (ii) –*x*+1, *y*+1/2, -*z*; (iii) –*x*, *y*-1/2, -*z*.
