# Supplementary figures and images for: Crystallographic study of PET radio­tracers in clinical evaluation for early diagnosis of Alzheimers
Source: Acta Crystallogr Sect E Struct Rep Online. 2014 Oct 4;70(Pt 11):o1149–50. doi: 10.1107/S1600536814021400 (PMC4257311; doi:10.1107/S1600536814021400)

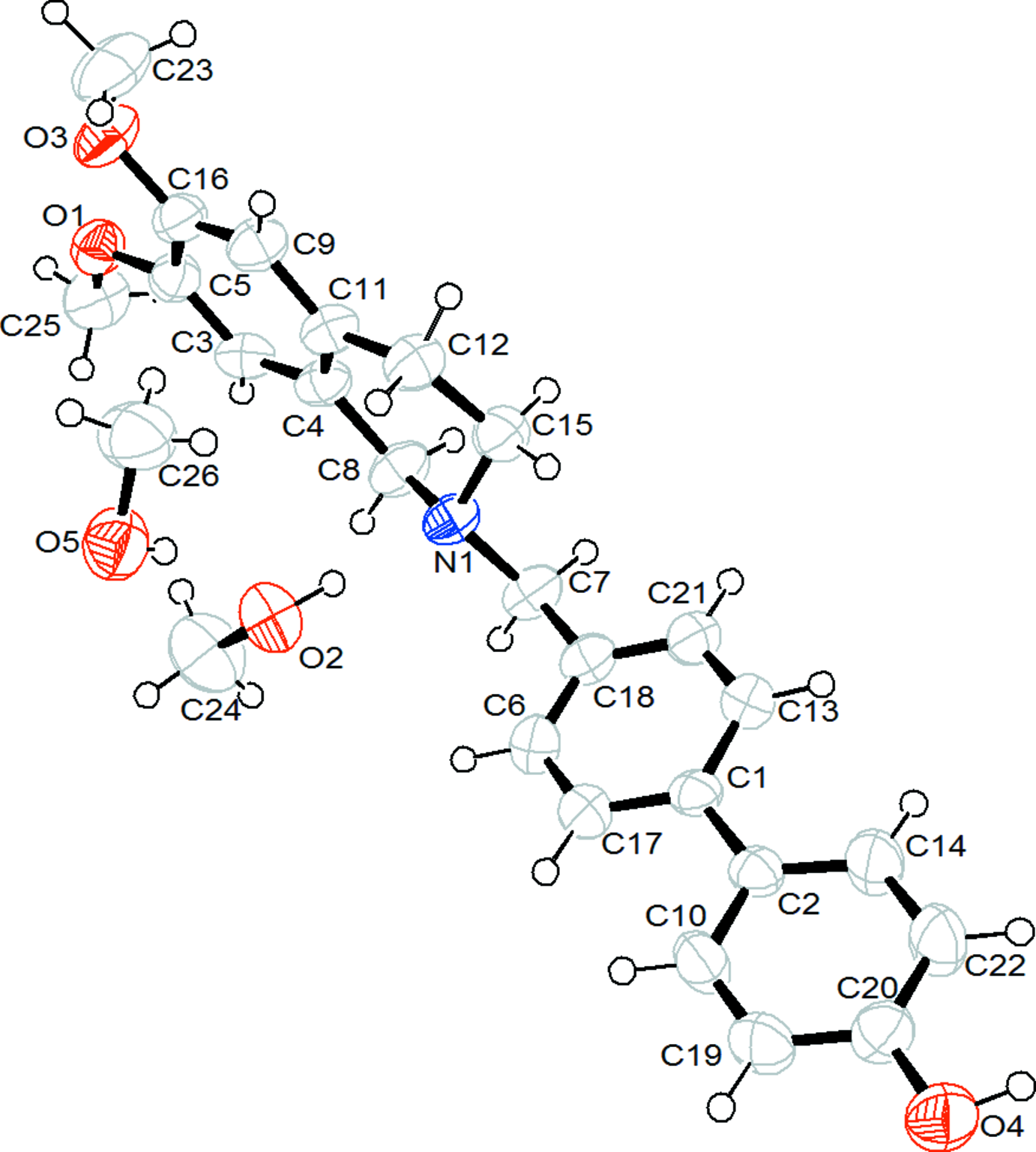

Supplement: Supplementary file 4 [file e-70-o1149-fig1.tif]

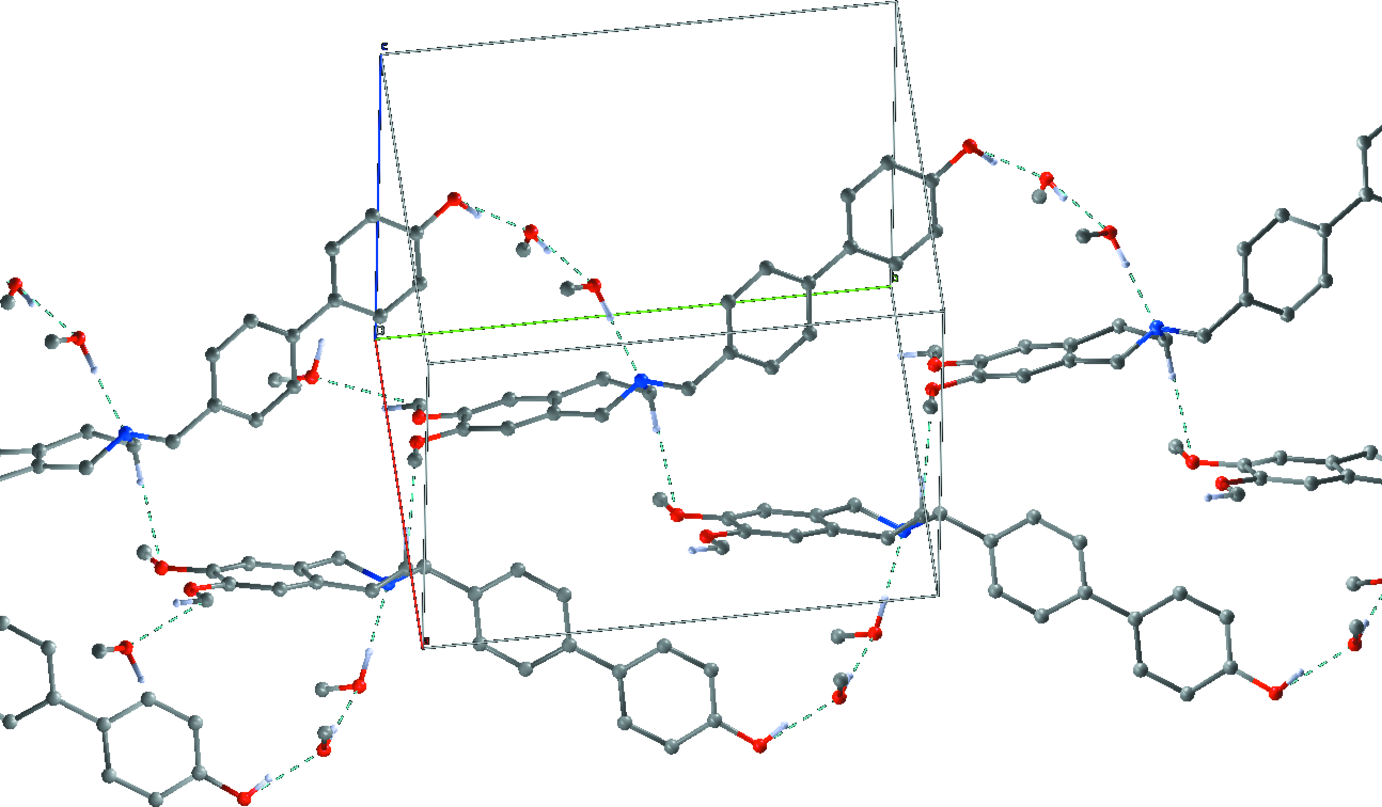

Supplement: Supplementary file 5 [file e-70-o1149-fig2.tif]
